# Direct In Vivo Comparison of ^99m^Tc-Labeled Scaffold Proteins, DARPin G3 and ADAPT6, for Visualization of HER2 Expression and Monitoring of Early Response for Trastuzumab Therapy

**DOI:** 10.3390/ijms232315181

**Published:** 2022-12-02

**Authors:** Vladimir Tolmachev, Vitalina Bodenko, Maryam Oroujeni, Sergey Deyev, Elena Konovalova, Alexey Schulga, Sarah Lindbo, Sophia Hober, Olga Bragina, Anna Orlova, Anzhelika Vorobyeva

**Affiliations:** 1 Department of Immunology, Genetics and Pathology, Uppsala University, 752 37 Uppsala, Sweden; 2 Research Centrum for Oncotheranostics, Research School of Chemistry and Applied Biomedical Sciences, Tomsk Polytechnic University, 634050 Tomsk, Russia; 3 Affibody AB, 171 65 Solna, Sweden; 4 Molecular Immunology Laboratory, Shemyakin-Ovchinnikov Institute of Bioorganic Chemistry, Russian Academy of Sciences, 117997 Moscow, Russia; 5 Department of Protein Science, KTH Royal Institute of Technology, 100 44 Stockholm, Sweden; 6 Department of Nuclear Medicine, Cancer Research Institute, Tomsk National Research Medical Center, Russian Academy of Sciences, 634009 Tomsk, Russia; 7 Department of Medicinal Chemistry, Uppsala University, 751 23 Uppsala, Sweden

**Keywords:** radionuclide molecular imaging, HER2, scaffold proteins, DARPin, ADAPT6, technetium-99m, preclinical

## Abstract

Non-invasive radionuclide molecular visualization of human epidermal growth factor receptor type 2 (HER2) can provide stratification of patients for HER2-targeting therapy. This method can also enable monitoring of the response to such therapies, thereby making treatment personalized and more efficient. Clinical evaluation in a phase I study demonstrated that injections of two scaffold protein-based imaging probes, [^99m^Tc]Tc-(HE)_3_-G3 and [^99m^Tc]Tc-ADAPT6, are safe, well-tolerated and cause a low level of radioactivity in healthy tissue. The goal of this preclinical study was to select the best probe for stratification of patients and response monitoring. Biodistribution of both tracers was compared in mice bearing SKOV-3 xenografts with high HER2 expression or MDA-MB-468 xenografts with very low expression. Changes in accumulation of the probes in SKOV-3 tumors 24 h after injection of trastuzumab were evaluated. Both [^99m^Tc]Tc-ADAPT6 and [^99m^Tc]Tc-(HE)_3_-G3 permitted high contrast imaging of HER2-expressing tumors and a clear discrimination between tumors with high and low HER2 expression. However, [^99m^Tc]Tc-ADAPT6 has better preconditions for higher sensitivity and specificity of stratification. On the other hand, [^99m^Tc]Tc-(HE)_3_-G3 is capable of detecting the decrease of HER2 expression on response to trastuzumab therapy only 24 h after injection of the loading dose. This indicates that the [^99m^Tc]Tc-(HE)_3_-G3 tracer would be better for monitoring early response to such treatment. The results of this study should be considered in planning of further clinical development of HER2 imaging probes.

## 1. Introduction

Application of radionuclide molecular imaging for non-invasive measurement of predictive and pharmacodynamic biomarkers offers an opportunity to make treatment of metastatic and locally advanced malignancies more efficient [1]. An obvious objective for such measurement is the level of expression of human epidermal growth factor receptor type 2 (HER2 or ErbB2) [2,3]. Overexpression of this proto-oncogene contributes to a malignant transformation and is associated with an aggressive course of a number of carcinomas [4]. Targeted therapies, which are based on molecular recognition of HER2, suppress tumor growth and improve overall survival. The use of such therapeutics has become a standard of care for HER2-expressing cancers of breast [5], stomach and gastroesophageal junction [6]. Furthermore, targeting of HER2 is under clinical evaluation for therapy of carcinomas of lung [7], ovary [8] and uterus [9]. The major issues in treatment of HER2 are the possible intertumoral heterogeneous expression in the primary tumor as well as in multiple metastases [10,11] and the change of HER2 expression level during the courses of disease treatment [12]. Both these phenomena might result in a serious undertreatment of patients. The invasive character of the biopsy-based methods significantly reduces the possibility of sampling and determining the expression of HER2 in multiple metastases, as well as evaluating its expression dynamics during chemo/targeted therapy. The noninvasiveness of radionuclide imaging makes it possible to solve these problems.

A wide spectrum of HER2-specific targeting probes, which ranged from full-length IgG to short peptides, has been evaluated for radionuclide imaging of HER2 [2]. Comparison of preclinical and clinical data shows that small, high affinity binders, such as single domain antibodies or engineered scaffold proteins, possibly are the preferable types of imaging agents [13]. Single-domain antibodies (sdAb, Nanobody) are the smallest targeting agents (molecular weight 12–15 kDa) based on immunoglobulins and consist of the variable domain of a heavy-chain camelid antibody (V_H_H) [14]. Engineered scaffold proteins utilize non-immunoglobulin scaffolds to ensure structural stability, which is required for selection of high-affinity binders [15]. The use of non-immunoglobulin scaffolds enables selection of small binders with a molecular weight in the range between 4 and 19 kDa and affinities below 5 nM. Unlike full-length IgG, such agents enable high-contrast imaging within 2–4 h after injection [1,2,16]. Several imaging probes, including ^99m^Tc- and ^68^Ga-labeled single-domain antibodies [17,18], ^111^In- and ^68^Ga-labeled affibody molecules [19,20], ^99m^Tc-labeled ADAPT6 [21] and DARPin G3 [22], have been evaluated in clinical trials. These studies have demonstrated that such probes are well-tolerated, not associated with any measurable toxicity and ensure a low absorbed dose burden for patients. This creates a demand to proceed to further clinical evaluations. It is essential to select an optimal targeted probe combined with an optimal radionuclide for this purpose. The recent development in single-photon emission computed tomography (SPECT) instrumentation, including the SPECT/CT combination as well as the use of semiconductor detectors, has addressed problems of absolute quantification of activity in vivo and localization of activity accumulation foci. Thereby, SPECT has become highly suitable for quantitative molecular imaging [23]. At the same time, SPECT-based diagnostic facilities are appreciably cheaper than facilities based on positron-emission tomography (PET) and thus. more available outside developed countries (see e.g., [24]). Furthermore, the use of a generator-produced radionuclide ^99m^Tc provides SPECT-based facilities with tracers even if delivery logistics are challenging. Thus, development of molecular imaging probes, which are suitable for SPECT imaging, might have a broad impact on global healthcare in the future.

**Figure 1:**
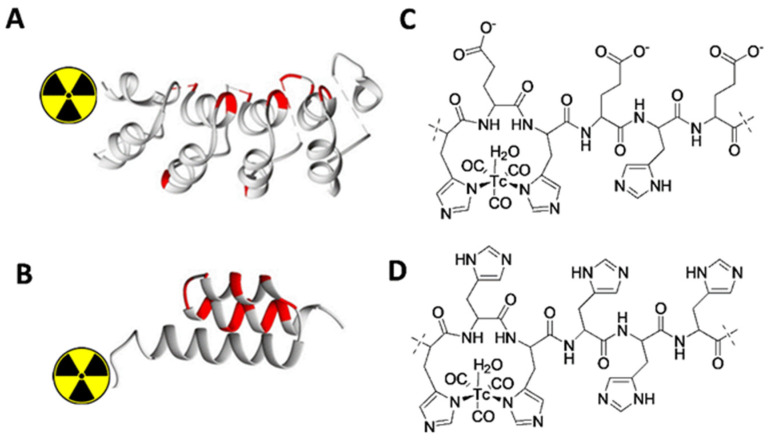
Schematic presentations of the three dimensional structures of the scaffolds (**A**,**B**) and labels (**C**,**D**) for [^99m^Tc]Tc-(HE)_3_-G3 (**A**,**C**) and [^99m^Tc]Tc-ADAPT6 (**B**,**D**).

Phase I clinical studies have shown that ^99m^Tc-labeled ADAPT6 [21] and DARPin G3 [22] provide discrimination between HER2-positive and HER2-negative breast cancer tumors and are potential candidates for further clinical development. These imaging probes are based on very different scaffolds (Figure 1). DARPin G3 is composed of four repeats based on a helix-turn-helix motif (Figure 1A), while the scaffold of ADAPT6 is based on a single three-helix bundle (Figure 1B). The probes bind to different epitopes on the extracellular domain of HER2. ADAPT6 binds to the same region as trastuzumab (on subdomain IV, close to C-terminus of the extracellular domain), and its binding is blocked by trastuzumab in vitro [25] and in vivo [26]. DARPin G3 binds also to subdomain IV, but close to its N-terminus and its epitope is different from the epitope of trastuzumab [27]. Labeling of both imaging agents is based on the use of technetium tricarbonyl core ([^99m^Tc]Tc(CO)_3_^+^) produced using a commercially available kit (CRS, Paul Scherrer Institute, Villigen, Switzerland); however, different peptide-based chelators are used. Preclinical studies have shown that the best chelator for ADAPT6 is the hexahistidine-tag (Figure 1C) placed at the N-terminus [26]. In the case of the DARPin, the optimal chelator was shown to be the peptide His-Glu-His-Glu-His-Glu ((HE)_3_-tag) (Figure 1D) [28]. Placement of this chelator at the N-terminus enabled three-fold reduction of the liver uptake of ^99m^Tc-labeled DARPin G3 compared with the hepatic uptake of the variants labeled using the hexahistidine tag [28].

Ideally, a tracer should provide data to enable selection of patients for targeted therapy and to monitor the response of such therapy. For stratification, sensitivity and specificity are the most important features. Sensitivity depends on the tracer uptake in the tumor compared to typical metastatic sites. For cancers with potentially high HER2 expression (breast, gastric, esophageal and ovarian), metastases are often formed in bone, liver, lung and peritoneal organs [29,30,31,32]. Accordingly, high ratios of uptakes in tumors compared to healthy tissues in these organs are essential to reach high sensitivity imaging. Specificity depends on the difference between the uptake in tumors with high expression, which are suitable for HER2-targeting therapy and the tumors with low expression. The higher the ratio that is achieved, the easier it is to discriminate between clinically HER2-positive and HER2-negative tumors. In addition, uptake in HER2-possitive lesions should be as high as possible to ensure a short scanning time and low dose burden to patients. It has to be noted that the clinical evaluation of [^99m^Tc]Tc-(HE)_3_-G3 and [^99m^Tc]Tc-ADAPT6 was performed on a stand-alone SPECT scanner, and therefore, the exact absolute quantification of uptake values for comparison is complicated. In addition, the preclinical characterization was performed three years apart, and a batch-to-batch variability in mice physiology and xenograft features make an exact comparison difficult. Therefore, it would be desirable to perform a comparison between these tracers directly, in the same batch of mice bearing xenografts developed from cells of the same passage.

Another important issue in personalized treatment of cancer is to have the ability to monitor the therapeutic response on a molecular level. Imaging using ^111^In-labeled trastuzumab during trastuzumab therapy demonstrated that the tumor uptake decreased by approximately 20% between day 1 of cycle 1 and day 15 of cycle 4 [33]. The authors concluded that a certain number of HER2 receptors is constantly available at the tumor cell membrane and that it is impossible to saturate the receptors completely. More likely, the response monitoring should be based on a measurement of expression levels. Preclinical studies indicated that decrease of the HER2 expression level is an early event, which starts 24–48 h after a treatment initiation [34,35]. Both these studies involved HER2-targeting agents that bind to a different epitope than trastuzumab, ^111^In-labeled pertuzumab, which binds to domain II of HER2 and an affibody molecule that binds to a different epitope on the domain III [36]. Binding to a different epitope than trastuzumab is essential in this context, as the imaging probe should enable discrimination between the decrease in binding because of epitope blocking or because of internalization/downregulation of the receptor. DARPin G3 meets this requirement.

The aim of this study was to perform a direct comparison between [^99m^Tc]Tc-(HE)_3_-G3 and [^99m^Tc]Tc-ADAPT6 in the same batch of immunodeficient mice bearing HER2-expressing xenografts. Another goal was to evaluate if [^99m^Tc]Tc-(HE)_3_-G3 can be used for early detection of downregulation of HER2 expression within 24 h after injection of a clinically relevant dose of trastuzumab.

## 2. Results

### 2.1. Protein Production and Labeling

Applied methodology permitted an efficient recombinant production of both (HE)_3_-G3 and ADAPT6 using simple procedures and IMAC purification. ESI-MS confirmed that the measured molecular weight of both proteins coincided with the calculated one (the difference within 1 Da). Reversed phase high performance liquid chromatography has shown that the purity of both proteins was above 95%.

The radiochemical yield was 96.8 ± 0.2 and 83 ± 8% for [^99m^Tc]Tc-ADAPT6 and [^99m^Tc]Tc-(HE)_3_-G3, respectively. The radiochemical purity after NAP-5 purification was 96.8 ± 0.2 and 83 ± 8% for [^99m^Tc]Tc-ADAPT6 and [^99m^Tc]Tc-(HE)_3_-G3, respectively. The maximal molar activity was 20.9 GBq/µmol for [^99m^Tc]Tc-ADAPT6 and 54.6 GBq/µmol for [^99m^Tc]Tc-(HE)_3_-G3.

### 2.2. In Vitro Binding and Saturation Experiments

**Figure 2:**
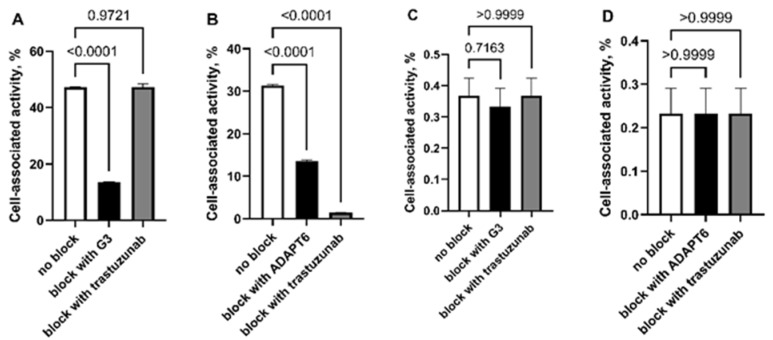
Effect of HER2 saturation on binding of [^99m^Tc]Tc-(HE)_3_-G3 (**A**,**C**) and [^99m^Tc]Tc-ADAPT6 (**B**,**D**) to human cancer cell lines in vitro. The tested cell lines were SKOV-3 with high HER2 expression (**A**,**B**) and MDA-MB-468 with low HER2 expression (**C**,**D**). Data are presented as a mean values and standard deviations for three samples. *p* value was determined by a one-way ANOVA with Dunnett correction for multiple comparisons.

The results of the in vitro binding and saturation experiments are presented in Figure 2, where a large difference between the patterns of binding of the different probes to HER2-positive SKOV3 and HER2-negative MDA-MB-468 cells in vitro. First and foremost, the percentage of activity associated with HER2-positive SKOV-3 cells (33.3 ± 0.3% for [^99m^Tc]Tc-ADAPT6 and 47.2 ± 0.1% for [^99m^Tc]Tc-(HE)3-G3) was significantly (*p* < 5 × 10^−9^, unpaired *t*-test) higher than the activity associated with the equal number of HER2-negative MDA-MB-468 cells (0.23 ± 0.06% for [^99m^Tc]Tc-ADAPT6 and 0.37 ± 0.06% for [^99m^Tc]Tc-(HE)_3_-G3).

In the case of the SKOV-3 cell line, with high HER2 expression, a saturation of HER2 with an excess of unlabeled proteins resulted in a highly significant (*p* < 0.0001 in unpaired *t*-test) reduction of binding of both proteins. In the case of HER2-negative MDA-MB-468 cells, adding the same excess of unlabeled protein did not influence the percentage of cell-associated activity. Adding trastuzumab during the experiment had no effect on [^99m^Tc]Tc-(HE)_3_-G3 binding to SKOV-3 cells but significantly (more than 20-fold, *p* < 0.0001 in unpaired *t*-test) reduced the binding of [^99m^Tc]Tc-ADAPT6 to this cell line.

### 2.3. Measurement of [^99m^Tc]Tc-(HE)_3_-G3 Affinity to HER2 in the Presence of Trastuzumab

**Figure 3:**
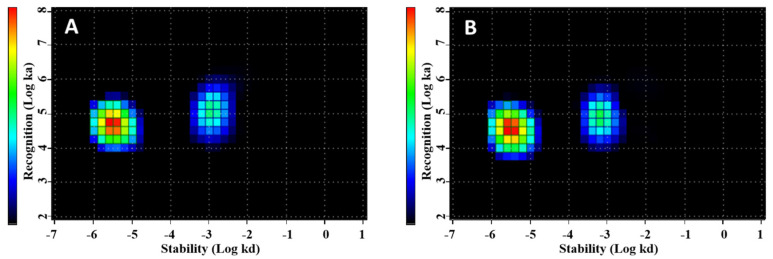
Graphic presentation of InteractionMap analysis of interaction of [^99m^Tc]Tc-(HE)_3_-G3 with SKOV-3 cells in vitro in the presence (**A**) or absence (**B**) of added trastuzumab.

The results of an InteractionMap analysis [37] of LigandTracer data concerning kinetics of [^99m^Tc]Tc-(HE)_3_-G3 interaction with living SKOV3 cells are presented in Figure 3 and Table 1. The data show two major interactions of the labeled protein with the receptors on living cells. A predominant (50–55% of all interactions) interaction had an affinity of approximately 100 pM. The second most abundant interaction (30%) had 9–10 nM affinity. Both interactions had similar association rates but the second interaction had a faster dissociation rate. There was no significant difference (*p* > 0.05, unpaired *t*-test) between dissociation constants in the presence or absence of trastuzumab.

### 2.4. In Vivo Experiments

**Figure 4:**
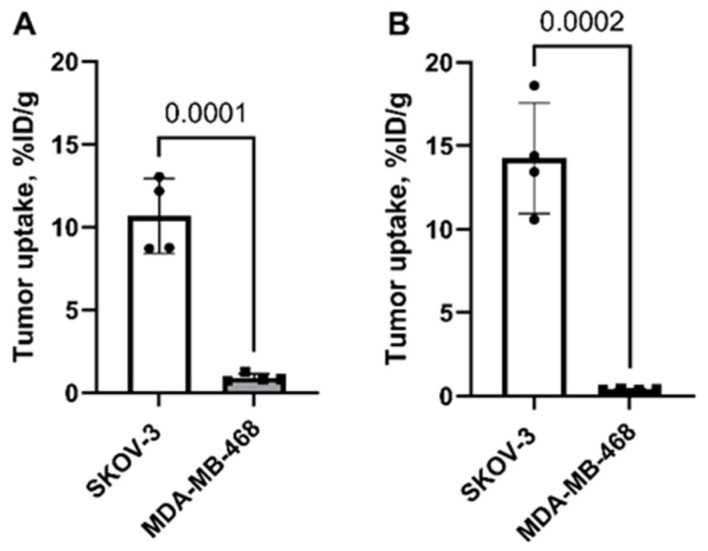
Comparison of uptake of [^99m^Tc]Tc-(HE)_3_-G3 (**A**) and [^99m^Tc]Tc-ADAPT6 (**B**) in xenografts with high HER2 expression (SKOV-3) and low HER2 expression (MDA-MB-468) in nude mice. Data are presented as mean values and standard deviations for four mice. *p* value is determined by an unpaired *t*-test.

Both [^99m^Tc]Tc-ADAPT6 and [^99m^Tc]Tc-(HE)_3_-G3 had significantly (*p* <0.005, unpaired *t*-test) higher uptake in HER2-positive SKOV-3 than in HER2-negative MDA-MB-468 tumors (Figure 4). The uptake of [^99m^Tc]Tc-ADAPT6 was 36-fold higher in SKOV-3 than in MDA-MB-468 (Figure 4B) and the difference was only 12-fold for [^99m^Tc]Tc-(HE)_3_-G3 (Figure 4A). The difference between the uptake of each tracer in other organs was not significant (*p* > 0.05, unpaired *t*-test) nor between uptakes of [^99m^Tc]Tc-(HE)_3_-G3 and [^99m^Tc]Tc-ADAPT6 in SKOV-3 tumors (*p* > 0.05, unpaired *t*-test).

**Figure 5:**
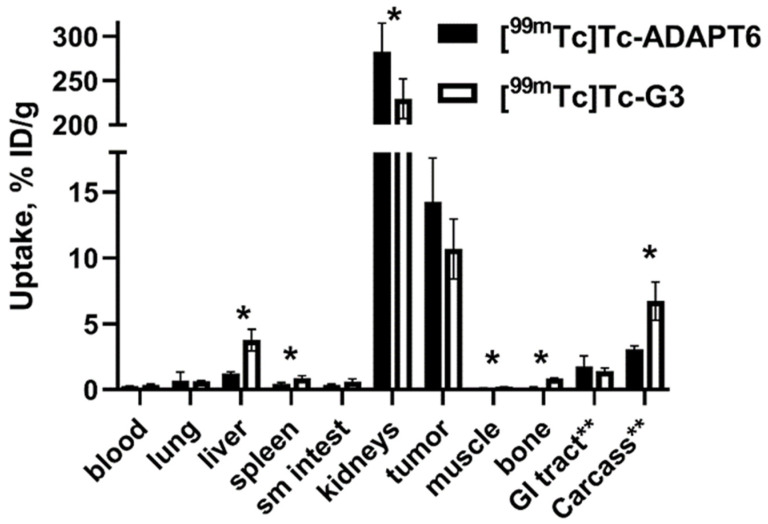
Comparison of biodistribution of [^99m^Tc]Tc-(HE)_3_-G3 and [^99m^Tc]Tc-ADAPT6 in mice bearing SKOV-3 xenografts with high HER2 expression. Data are presented as mean values and standard deviations for four mice. * Asterisks show significant difference between uptake of tracers (*p* < 0.05, unpaired *t*-test).

Comparison of the biodistribution of [^99m^Tc]Tc-ADAPT6 and [^99m^Tc]Tc-(HE)_3_-G3 in mice is shown in Figure 5. In agreement with the previous studies, both labeled scaffold proteins cleared rapidly from normal organs. The blood concentration at this time point was only 0.27 ± 0.2 and 0.35 ± 0.7% ID/g for [^99m^Tc]Tc-ADAPT6 and [^99m^Tc]Tc-(HE)_3_-G3, respectively (no significant difference, *p* > 0.05 in unpaired *t*-test). The activity in the whole gastrointestinal tract, including content, was 1.8 ± 0.8 and 1.4 ± 0.2% ID for [^99m^Tc]Tc-ADAPT6 and [^99m^Tc]Tc-(HE)_3_-G3, respectively (no significant difference, *p* > 0.05 in unpaired *t*-test). This shows that the hepatobiliary excretion did not play any substantial role in elimination of the tested compounds. Both compounds demonstrated high renal reabsorption, 283 ± 32% ID/g for [^99m^Tc]Tc-ADAPT6 and 229 ± 22% ID/g for [^99m^Tc]Tc-(HE)_3_-G3. The renal uptake of [^99m^Tc]Tc-ADAPT6 was significantly (*p* < 0.05, unpaired *t*-test) higher than the uptake of radiolabeled DARPin. At the same time, [^99m^Tc]Tc-ADAPT6 had significantly (*p* < 0.05, unpaired *t*-test) lower uptake in liver, spleen, muscle and bone and the retention of [^99m^Tc]Tc-(HE)_3_-G3 in the rest of the body was significantly (*p* < 0.05, unpaired *t*-test) higher.

**Figure 6:**
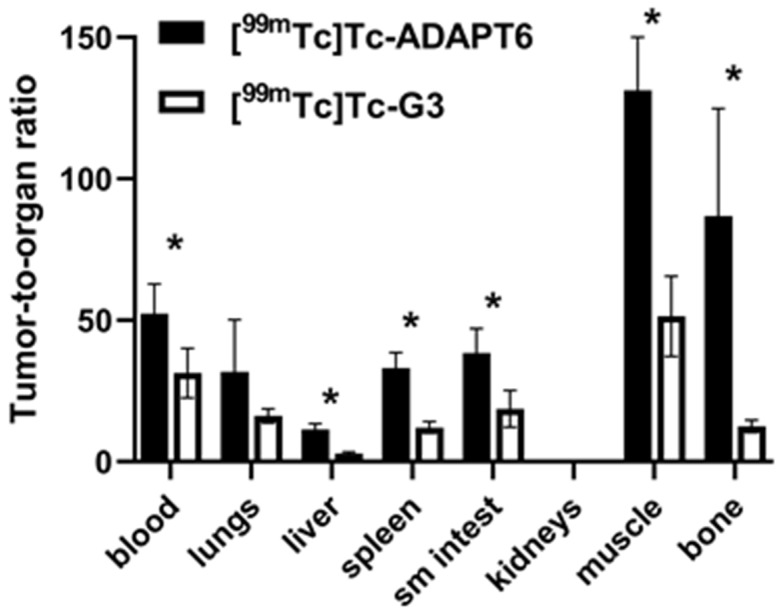
Comparison of tumor-to-organ ratios provided by [^99m^Tc]Tc-(HE)_3_-G3 and [^99m^Tc]Tc-ADAPT6 in nude mice with SKOV-3 xenografts with high HER2 expression 4 h after injection. Data are presented as a mean values and standard deviations for four mice. Asterisks show significant difference between uptake of tracers (*p* < 0.05, unpaired *t*-test).

Comparison of the tumor-to-organ ratios provided by [^99m^Tc]Tc-ADAPT6 and [^99m^Tc]Tc-(HE)_3_-G3 is shown in Figure 6. Tumor-to-blood, tumor-to-liver, tumor-to-spleen, tumor-to-small intestine, tumor-to-muscle and tumor-to-bone ratios were significantly (*p* < 0.05, unpaired *t*-test) higher for [^99m^Tc]Tc-ADAPT6.

**Figure 7:**
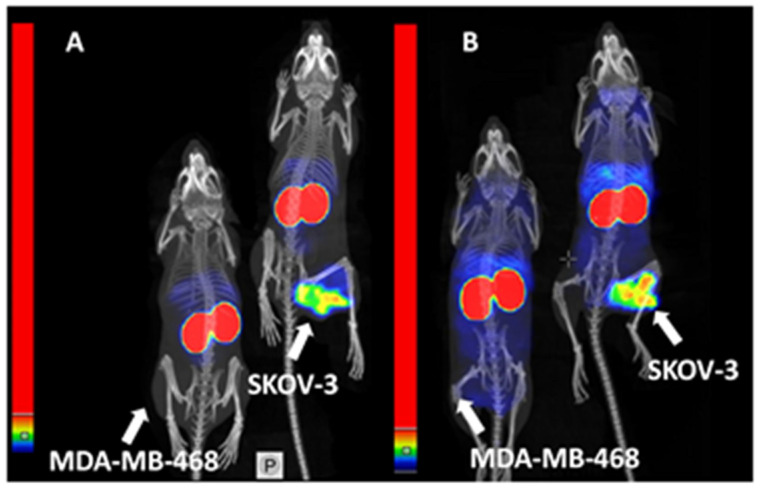
Imaging of nude mice bearing HER2-positive SKOV-3 or HER2-negative MDA-MB-468 xenografts 4 h after injection of [^99m^Tc]Tc-ADAPT6 (**A**) and [^99m^Tc]Tc-(HE)_3_-G3 (**B**). Scales were adjusted to show red pixels in SKOV-3 xenografts for each conjugate.

The results of experimental imaging (Figure 7) were in agreement with the biodistribution data. Both [^99m^Tc]Tc-ADAPT6 and [^99m^Tc]Tc-(HE)_3_-G3 had the highest accumulation in kidneys. A scale adjustment permitted a clear, high-contrast visualization of HER2-positive SKOV-3 tumors. With the same scale settings, HER2-negative MDA-MB-468 tumors were not visible. Accumulation of [^99m^Tc]Tc-ADAPT6 in liver in relation to its accumulation in tumor was lower compared with accumulation of [^99m^Tc]Tc-(HE)_3_-G3.

**Figure 8:**
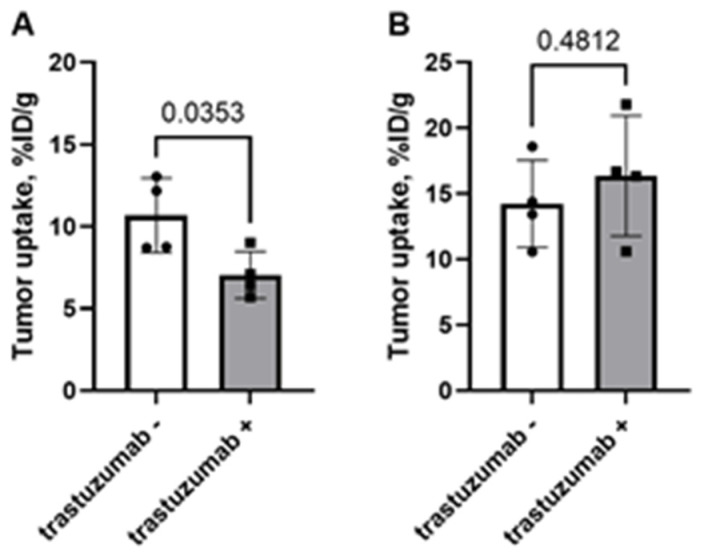
Uptake of [^99m^Tc]Tc-(HE)_3_-G3 (**A**) and [^99m^Tc]Tc-ADAPT6 (**B**) in SKOV-3 xenografts when trastuzumab was injected 24 h before injection of the tracer (trastuzumab+) or without preinjection of trastuzumab (trastuzumab-). Data are presented as mean values and standard deviations for four mice. *p* value is determined by an unpaired *t*-test.

A comparison of uptake of tracers in HER2-positive SKOV-3 tumors 24 h after injection of a loading dose of trastuzumab (4 mg/kg) is shown in Figure 8. A pretreatment with trastuzumab resulted in 1.5-fold reduction of [^99m^Tc]Tc-(HE)_3_-G3 uptake (*p* < 0.05, unpaired *t*-test). The tumor uptake of [^99m^Tc]Tc-ADAPT6 did not change significantly (*p* > 0.05, unpaired *t*-test). The preinjection did not change the uptake of any of the tracers in normal organs and tissues.

## 3. Discussion

For the moment, three ^99m^Tc-labeled HER2 imaging agents passed clinical evaluation [18,21,22]. The scaffold-protein-based agents [^99m^Tc]Tc-(HE)_3_-G3 and [^99m^Tc]Tc-ADAPT6 revealed the difference between clinically HER2-positive and HER2-negative breast cancer tumors [21,22]. In the case of ^99m^Tc-labeled sdAb NM-02 [18], a number of clinically HER2-negative tumors had higher SUV values than HER2-positive in the Phase I clinical study, i.e., the tumor uptake did not match the expression level of HER2. Thus, only [^99m^Tc]Tc-(HE)_3_-G3 and [^99m^Tc]Tc-ADAPT6 are promising candidates for further clinical development. Imaging of HER2 might have two applications in personalized medicine, stratification of patients for a HER2-specific targeted therapy and monitoring of response to this therapy. The design of future clinical studies depends on the clinical use. This study aimed to answer the question as whether any of the tracers would suit both applications or if the tracers should be different for each kind of diagnostics.

Selection of the best probes should take into consideration different aspects, both medical and economic. From an economic point of view, both DARPin G3 and ADAPT6 have a good precondition for production in developing countries. Both scaffold proteins can be efficiently produced in prokaryotic organisms, which reduces the cost of the goods. The use of histidine-containing tags permits straightforward, efficient and inexpensive purification using an immobilized metal ions affinity chromatography (IMAC) [38]. The use of the same tags as chelators for labeling by ^99m^Tc streamlines the production process further for both probes equally. Thus, only radiopharmacology considerations should determine the choice.

In vitro data from this study demonstrated higher binding of both tracers to HER2-positive than to HER2-negative cells, and saturable binding to HER2-positive cells (Figure 2). This confirmed that their binding in vitro is HER2-specific. However, the level of [^99m^Tc]Tc-(HE)_3_-G3 binding to HER2-positive SKOV-3 cells was higher, when cells were incubated with equal concentration of both probes. This correlates with the higher affinity of [^99m^Tc]Tc-(HE)_3_-G3 to HER2. Short-term incubation with trastuzumab in vitro did not reduce the binding of [^99m^Tc]Tc-(HE)_3_-G3 to SKOV-3 cells, but reduced the uptake of [^99m^Tc]Tc-ADAPT6, which was in agreement with previous data [26,28].

In earlier preclinical studies, the level of [^99m^Tc]Tc-ADAPT6 uptake in SKOV-3 xenografts 4 h, 19 ± 3% ID/g [26], was higher than the uptake of [^99m^Tc]Tc-(HE)_3_-G3, 9 ± 1% ID/g [28]. In this study, the tumor uptake was compared after injection of an equimolar amount of tracers in the same batch of mice with similar xenografts to minimize the variability between animals and implanted cells. This test did not find any significant difference between [^99m^Tc]Tc-(HE)_3_-G3 and [^99m^Tc]Tc-ADAPT6 uptakes in SKOV-3 tumors (*p* > 0.05, unpaired *t*-test) (Figure 4). Thus, the absolute tumor uptake level cannot be taken into consideration for selection of the probe for clinical applications. However, the uptake of [^99m^Tc]Tc-ADAPT6 was 36-fold higher in HER2-positive SKOV-3 tumors than in HER2-negative MDA-MB-468 while the difference between the uptake in these different tumors for [^99m^Tc]Tc-(HE)_3_-G3 was only 12-fold. Taking into account that the larger the difference between positive and negative tumors, the better the possibility is to discriminate between them in clinical settings, [^99m^Tc]Tc-ADAPT6 has higher potential for better specificity of an image-based diagnostics.

For high sensitivity imaging, an important precondition is high contrast, which is determined by both high uptake in tumor and low uptake in normal tissues. [^99m^Tc]Tc-ADAPT6 has significantly (*p* < 0.05, unpaired *t*-test) lower uptake in liver, spleen, small intestines, muscle and bone (Figure 5) than [^99m^Tc]Tc-(HE)_3_-G3, resulting in higher imaging contrast to these tissues (Figure 6). This feature should provide higher sensitivity of [^99m^Tc]Tc-ADAPT6 for visualization of metastases in liver, abdominal organs and bone, where HER2-expressing cancers often metastasize. The biodistribution data were confirmed by the results of the imaging experiment (Figure 7). This indicates that [^99m^Tc]Tc-ADAPT6 is preferred, compared to [^99m^Tc]Tc-(HE)_3_-G3, for stratification of patients for therapies, where sensitive discrimination between HER2-positive and HER2-negative tumors is essential.

Earlier studies have shown that a decrease of HER2 expression in tumors might occur as early as 24 h after initiation of trastuzumab treatment and this can be detected using radionuclide imaging [35]. Our previous data showed that the uptake of [^99m^Tc]Tc-ADAPT6 in HER2-expressing xenografts in mice might be completely blocked by pre-injection of trastuzumab [26]. However, these earlier experiments were performed using a large amount of the antibody, 10 mg per mouse, i.e., 500 mg/kg. This exceeds, by far, the clinical dosing (4 mg/kg for loading dose and 2 mg/kg thereafter). In this study, we used a clinically relevant dosing. Comparison of the effect of trastuzumab injection on the uptake of [^99m^Tc]Tc-(HE)_3_-G3 and [^99m^Tc]Tc-ADAPT6 was paradoxical: there was a significant decrease of the [^99m^Tc]Tc-(HE)_3_-G3 uptake in HER2-expressing SKOV-3 xenografts but no decrease of the [^99m^Tc]Tc-ADAPT6 uptake (Figure 8). This contradicts the in vitro blocking data (Figure 2) and results of the affinity measurements showing that binding of [^99m^Tc]Tc-(HE)_3_-G3 to HER2-expressing cells is not affected by a short-term exposure to trastuzumab (Figure 3, Table 1). However, these data might be explained by features like localization and extravasation of targeting proteins with different size and affinity into tumors. Monoclonal antibodies are bulky proteins (molecular weight of IgG is 150 kDa). This limits their permeability through the vascular endothelium and penetration into a tumor mass [39,40]. It might be expected that 24 h after injection of trastuzumab, only a few outer layers of cells in the tumor would be affected by the treatment, while the core of tumors would not be influenced. Since the molecular weight of DARPins (14.5 kDa) is higher than ADAPTs (7 kDa), DARPins would penetrate into tumors less efficiently. Another factor influencing penetration into tumor mass is the affinity to a molecular target. Penetration of targeting proteins with very high affinity is less efficient due to a so-called “binding site barrier” [41,42]. The affinity of [^99m^Tc]Tc-ADAPT6 to HER2 (2.8 nM [26]) to HER2 is, by more than one order of magnitude, lower than the affinity of [^99m^Tc]Tc-(HE)_3_-G3 (96 ± 3 pM [28] or 83 ± 28 pM (this study). Therefore, it might be expected that [^99m^Tc]Tc-ADAPT6 would penetrate deeper into tumors and reach the area where HER2 expression is still high. The more bulky, high affinity DARPin should remain in the outer layers of tumor nodules, where HER2 expression is reduced by the trastuzumab treatment, and thereby, their binding would be lower in comparison with the binding to non-treated tumor core. Apparently, this issue deserves further thorough investigations. However, it is clear that [^99m^Tc]Tc-(HE)_3_-G3 is more suitable for detection of pharmacodynamic effects of trastuzumab on HER2-expressing tumors shortly after the treatment initiation.

## 4. Materials and Methods

### 4.1. Protein Production and Labeling

(HE)_3_-G3 was produced according to the procedure described by Vorobyeva and co-workers [28] and ADAPT6 by the procedure described by Lindbo and co-workers [26]. Authenticity of the compounds was evaluated using electrospray ionization mass spectroscopy (ESI-MS) and liquid chromatography, respectively, as described in the original publications.

The labeling was performed according to procedures described earlier [26,28] with a slight modification, a pH adjustment of [^99m^Tc]Tc(CO)_3_^+^ solution. Briefly, 400 µL of eluate from a ^99m^Tc-generator (Ultra TechneKow, Mallinckrodt, Petten, The Netherlands) containing 900–1000 MBq ^99m^Tc was added to a vial containing CRS kit. The vial was incubated at 100 °C for 30 min, the solution containing 120–150 MBq was transferred to another vial and pH was adjusted with 0.1 M HCl (by adding an volume three-fold bigger than the volume of [^99m^Tc]Tc(CO)_3_^+^-containing solution).

For labeling of ADAPT6, 50 µg of protein in 25 µL phosphate-buffered saline (PBS), pH 7.5 was added to the [^99m^Tc]Tc(CO)_3_^+^-containing solution. The mixture was incubated at 50 °C for 30 min. Thereafter, 500-fold excess of L-histidine (10 mg/mL in PBS) was added, and the mixture was incubated at 50 °C for another 5 min. [^99m^Tc]Tc-ADAPT6 was purified using disposable NAP5 columns (Cytiva, Uppsala, Sweden).

For labeling of (HE)_3_-G3, 40 µg of protein in 22 µL PBS was added to the neutralized [^99m^Tc]Tc(CO)_3_^+^-containing solution. The mixture was incubated at 60 °C for 30 min. Thereafter, 500-fold excess of L-histidine (10 mg/mL in PBS) was added, and the mixture was incubated at 60 °C for another 5 min. [^99m^Tc]Tc-(HE)_3_-G3 was purified using disposable NAP5 columns (Cytiva, Uppsala, Sweden).

The radiochemical yield and radiochemical purity of labeled proteins was measured by instant thin-layer chromatography (iTLC) analysis using iTLC silica gel strips (Varian, Lake Forest, CA, USA). Phosphate buffered saline (PBS) was used as a mobile phase. In this system, radiolabeled proteins remain at the application point (R_f_ = 0) and any form of unbound ^99m^Tc migrates with the solvent front (R_f_ = 1). A quantitative measurement of activity distribution in the iTLC strips was performed using a Storage Phosphor System (CR-35 BIO Plus, Elysia-Raytest, Bietigheim-Bissingen, Germany) and AIDA Image Analysis software (Elysia-Raytest, Bietigheim-Bissingen, Germany). 

### 4.2. Cell Lines

HER2-positive SKOV3 (ovarian carcinoma) and HER2-negative MDA-MB-468 (triple negative breast carcinoma) cell lines were obtained from the American Type Culture Collection (Manassas, VA, USA). The cells were cultured in a humidified incubator with 5% CO_2_ at 37 °C in RPMI medium (Biochrom, Berlin, Germany) containing 10% fetal bovine serum (FBS) (Merck, Darmstadt, Germany), 2 mM L-glutamine, 100 IU/mL penicillin and 100 μg/mL streptomycin (all from Biochrom, Berlin, Germany).

### 4.3. In Vitro Binding Saturation Experiments

The in vitro saturation experiments were performed in order to a) evaluate if the binding of radiolabeled proteins to the selected cell lines can be saturated, i.e., is target-specific, and b) if the binding can be blocked by the HER2-specific therapeutic monoclonal antibody trastuzumab after the short incubation with this antibody.

Cells were seeded in the cell culture dishes (10^6^ cells per dish) one day before the experiments. All experiments were performed in triplicates. For each compound and cell line, three sets of cells culture dishes (three dishes each) containing seeded cells were used. The cell culture medium was aspirated. To one set of control dishes, a medium (500 µL) containing 100 nM of unlabeled proteins was added. To another set of control dishes, a medium (500 µL) containing 100 nM of trastuzumab was added. To the third set of dishes, a pure medium (500 µL) was added. The cells were incubated 1 h at 37 °C. After the incubation, a medium (500 µL) containing 2 nM of radiolabeled protein was added, and the cells were incubated 1 h at 37 °C. Thereafter, the medium with the radioactive solution was collected from each dish, the cells were washed with 1 mL pure medium and the solution was combined with the radioactive medium solution. The cells were lysed by adding 1 mL of 1 M NaOH followed by an incubation for 30 min at 37 °C, and cell lysates were collected. The cells were washed with 1 mL of NaOH and the solution was combined with the lysate solution. Activities of incubation medium and lysate were measured for each sample and the percent of cell-bound activity was calculated. Statistical significance in the difference of cell-bound activity for each experiment was determined using one-way ANOVA with Dunnett correction for multiple comparisons. In addition, the statistical significance of difference between bindings of each labeled protein to HER2-positive SKOV3 and HER2-negative MDA-MB-468 cells was analyzed using unpaired *t*-test.

### 4.4. Measurement of [^99m^Tc]Tc-(HE)_3_-G3 Affinity to HER2 in the Presence of Trastuzumab

The real time kinetics of [^99m^Tc]Tc-(HE)_3_-G3 interaction with living SKOV3 cells was measured using a LigandTracer (Ridgeview Instruments, Vänge, Sweden) using the protocol described earlier [37]. For the measurements, 1.5 × 10^6^ of SKOV-3 cells were seeded on a segment of a cell culture dish one day before measurement. Kinetics of binding to the cells were measured at [^99m^Tc]Tc-(HE)_3_-G3 concentrations 0.5 and 2 nM followed by measurements of dissociation kinetics. The measurements were performed at the room temperature. All measurements were performed in triplicates in the absence of trastuzumab. In another series of experiments (triplicate), trastuzumab (100 nM) was added simultaneously with the addition of [^99m^Tc]Tc-(HE)_3_-G3, and further measurements were performed in the presence of trastuzumab using the same protocol. InteractionMap software (Ridgeview Diagnostics AB, Vänge, Sweden) was used to analyze the data in more detail. An unpaired *t*-test was used to determine if there is a statistically significant difference between equilibrium dissociation constants of [^99m^Tc]Tc-(HE)_3_-G3 interaction with SKOV-3 cells in the presence or absence of trastuzumab. Analysis of binding of [^99m^Tc]Tc-ADAPT6 was not performed because the saturation experiments (see above) showed clearly that its interaction with SKOV-3 cells is suppressed by adding trastuzumab.

### 4.5. In Vivo Experiments

The described procedures were reviewed and approved by the Animal Research Committee at Uppsala University (ethical permission 4/16) and were performed in accordance with the Swedish national legislation on the protection of laboratory animals. After tumor implantation, the tumor size and animal behavior were monitored twice a week. For implantation of tumors, 10^7^ of HER2-positive SKOV-3 cells or 10^7^ of HER2-negative MDA-MB-468 cells in 100 µL of media were subcutaneously injected in the hind legs of female BALB/c nu/nu mice (8 weeks old). The experiments were performed two weeks after implantation. The average animal weight was 19 ± 1 g in the SKOV3 groups and 19 ± 1 g in the MDA-MB-468 groups. The average tumor weight at the time of experiment was 0.07 ± 0.04 g for SKOV3 xenografts and 0.04 ± 0.03 g for MDA-MB-468 xenografts (no significant difference, *p* > 0.05 in unpaired *t*-test, between tumor sizes). A group of four mice was used per data point.

The mice were intravenously (tail vein) injected with either [^99m^Tc]Tc-(HE)_3_-G3 (40 kBq, 5 µg/0.35 nmol per mouse) or [^99m^Tc]Tc-ADAPT6 (40 kBq, 2.4 µg/0.35 nmol per mouse) in 100 μL of 1% BSA in PBS. The biodistribution was measured 4 h post injection (pi), which is a clinically relevant time point. The mice were anesthetized by an intraperitoneal injection of Ketalar and Rompun solution (lethal dose, ketamine (Ketalar, Pfizer, New York, NY, USA), 200 mg/kg of body weight, and xylazin (Rompun, Bayer, Leverkusen, Germany), 20 mg/kg of body weight) and sacrificed by heart puncture. Blood was collected with a heparinized syringe, organs were excised, weighed and activity of samples was measured using an automated gamma-spectrometer 1480 Wizard (Wallac, Finland). The measurements were corrected for decay and background. The percent of injected dose per gram of sample (%ID/g) was calculated. Statistical analysis was performed using GraphPad Prism software (GraphPad Software Inc., La Jolla, CA, USA).

Additionally, two groups of mice bearing SKOV-3 xenografts were injected with a clinically relevant dose of trastuzumab, 4 mg/kg body weight) 24 h before injection of [^99m^Tc]Tc-(HE)_3_-G3 or [^99m^Tc]Tc-ADAPT6 to evaluate if these tracers can be used for monitoring of early response to trastuzumab therapy. Thereafter, the biodistribution was measured as it is described above.

Imaging experiments were performed using a nanoScan SPECT/CT scanner (Mediso Medical Imaging Systems, Budapest, Hungary). Animals were injected with either [^99m^Tc]Tc-(HE)_3_-G3 (11 MBq, 5 µg/0.35 nmol per mouse) or [^99m^Tc]Tc-ADAPT6 (6 MBq, 2.4 µg/0.35 nmol per mouse). For each tracer, one mouse with SKOV-3 xenograft and one mouse with MDA-MB-468 xenograft were used to check in vivo specificity. Imaging was performed 4 h after injection. Immediately before imaging, the animals were euthanized by CO_2_ asphyxiation (displacement rate 35% per minute), which caused urination and removal of interfering activity from bladders. A mouse with SKOV-3 and a mouse with MDA-MB-468 xenograft were simultaneously placed in a prone position in the camera, and CT (50 keV, 670 μA, 5 min acquisition time) were acquired. Thereafter, an acquisition of SPECT scans (window from 126.45 keV to 154.56 keV, 256 × 256 matrix, 15 min acquisition time) was performed. The CT raw data were reconstructed using Nucline 2.03 Software (Mediso Medical Imaging Systems, Hungary). SPECT raw data were reconstructed using Tera-Tomo™ 3D SPECT.

## 5. Conclusions

The tumor uptake of [^99m^Tc]Tc-ADAPT6 and [^99m^Tc]Tc-(HE)_3_-G3 does not differ significantly. While both [^99m^Tc]Tc-ADAPT6 and [^99m^Tc]Tc-(HE)_3_-G3 permit high contrast imaging of HER2-expressing tumors and clear discrimination between tumors with high and low HER2 expression, the difference between uptake in tumors with high and low expression is bigger for [^99m^Tc]Tc-ADAPT6. This is a precondition for higher specificity of imaging using this tracer. In addition, this imaging probe has higher ratios between accumulation in tumors and organs, where HER2-expressing tumors often form metastases. This should result in higher sensitivity. Thus, [^99m^Tc]Tc-ADAPT6 would be the preferred tracer for stratification of patients for HER2-targeting therapies. On the other hand, [^99m^Tc]Tc-(HE)_3_-G3 is capable of sensing the decrease of HER2 expression on response to trastuzumab therapy only 24 h after injection of a therapeutic dose of this antibody. This indicates that this tracer would be better for monitoring early response to such treatment. Accordingly, further clinical development should be planned taking into account the results of this study.

## Figures and Tables

**Table 1:** Affinity of [^99m^Tc]Tc-(HE)_3_-G3 binding to SKOV-3 cells in vitro. The data are presented as an average and standard deviation for results of three independent LigandTracer experiments.

Measurement Condition	K_D1_ (pM)	Weight (%)	K_D2_ (nM)	Weight (%)
No addition of trastuzumab	83 ± 28	55 ± 3	9 ± 1	27 ± 2
With addition of trastuzumab	105 ± 29	51 ± 3	51 ± 3	31 ± 3

## Data Availability

All data are contained within the manuscript.
